# Spatial Navigation Is Distinctively Impaired in Persistent Postural Perceptual Dizziness

**DOI:** 10.3389/fneur.2019.01361

**Published:** 2020-01-09

**Authors:** Hayo A. Breinbauer, Maria Daniela Contreras, Juan P. Lira, Claudia Guevara, Leslie Castillo, Katherine Ruëdlinger, Daniel Muñoz, Paul H. Delano

**Affiliations:** ^1^Department of Otolaryngology, Facultad de Medicina, Universidad de Chile, Santiago, Chile; ^2^Department of Neurocience, Facultad de Medicina, Universidad de Chile, Santiago, Chile; ^3^Department of Otolaryngology, Facultad de Medicina Clínica Alemana, Universidad del Desarrollo, Santiago, Chile

**Keywords:** dizziness, functional dizziness, persistent postural perceptual dizziness, spatial navigation, morris water maze

## Abstract

**Objective:** To determine whether performance in a virtual spatial navigational task is poorer in persistent postural perceptual dizziness (PPPD) patients than in healthy volunteers and patients suffering other vestibular disorders.

**Methods:** Subjects were asked to perform three virtual Morris water maze spatial navigational tasks: (i) with a visible target, (ii) then with an invisible target and a fixed starting position, and finally (iii) with an invisible target and random initial position. Data were analyzed using the cumulative search error (CSE) index.

**Results:** While all subjects performed equally well with a visible target, the patients with PPPD (*n* = 19) performed poorer (*p* < 0.004) in the invisible target/navigationally demanding tasks (CSE median of 8) than did the healthy controls (*n* = 18; CSE: 3) and vestibular controls (*n* = 19; CSE: 4). Navigational performance in the most challenging setting allowed us to discriminate PPPD patients from controls with an area under the receiver operating characteristic curve of 0.83 (sensitivity 78.1%; specificity 83.3%). PPPD patients manifested more chaotic and disorganized search strategies, with more dispersion in the navigational pool than those of the non-PPPD groups (standard distance deviation of 0.97 vs. 0.46 in vestibular controls and 0.20 in healthy controls; *p* < 0.001).

**Conclusions:** While all patients suffering a vestibular disorder had poorer navigational abilities than healthy controls did, patients with PPPD showed the worst performance, to the point that this variable allowed the discrimination of PPPD from non-PPPD patients. This distinct impairment in spatial navigation abilities offers new insights into PPPD pathophysiology and may also represent a new biomarker for diagnosing this entity.

## Introduction

Persistent postural perceptual dizziness (PPPD) is a clinical entity that comprises different types of non-vertiginous dizziness and represents the most common cause of chronic vestibular syndromes (1, 2). There are no objective biomarkers for PPPD, and its diagnosis depends entirely on clinical criteria (1).

The physiopathology of PPPD remains unclear (2–5). One theoretical approach focuses on errors in visual, proprioceptive, and vestibular integration (6–8) and the reduced cortical integration of spatial orientation cues as a pathological response to a triggering event (9, 10). These disturbed computations of multimodal inputs can generate an inappropriate inner spatial model of the environment and of the patient's position in it. It has been proposed that the discrepancies between the inner spatial model and reality are responsible for PPPD symptomatology (2, 4, 11). This framework is particularly interesting, as it offers a better understanding of visual induced dizziness and symptoms reported in PPPD, particularly in regard to the response of complex visual stimuli or the difficulty these patient refer when being in crowed spaces, such as a mall or in a subway station, with multiple objects moving rapidly in different directions (which could be understood as an overload of multimodal information to be processed, and a subsequent difficulty on constructing an accurate and properly timed inner model).

However, whether an altered internal cognitive map of the spatial environment is distinctively impaired in PPPD patients compared with individuals with other non-PPPD vestibular pathologies remains unknown (6, 12, 13).

We hypothesize that an important feature of PPPD is a disturbance in the mechanisms necessary to maintain an internal spatial map of the environment, which can impact individuals' performance in a navigational task.

Spatial navigation, an ability that emerges from the proper management of an internal spatial map, can be tested by means of the Morris water maze (MWM) experimental paradigm (14), in which the subject (originally a rodent) swims freely in a round pool that has visual cues installed around it. A transparent glass platform, which the subject cannot initially see, is located in the pool. The subject must find the platform to rest. He must also remember its location in relation to the visual cues to reach it faster on subsequent trials of the test by following a more direct route. Subjects with impaired memory or spatial navigation abilities (such as hippocampal lesions) fail to locate the platform and wander erratically around for a long period of time, even after having found the platform in previous trials. For human testing, virtual versions of the MWM paradigm have been validated and proved for use in identifying memory impairments, such as those found in individuals with Alzheimer's disease (15–18).

Navigational abilities have been explored in vestibular research, having found decreased performance in navigation in bilateral peripheral vestibular loss (12, 19). Nevertheless, the interpretation of these findings has been questioned due to methodological issues and revisited (13, 20), suggesting that there might be confounding variables involved, regarding cognitive and emotional aspects of patients. We believe that the confounding variable might indeed be the presence of PPPD.

Here, we studied spatial navigation in patients with PPPD, vestibular patients without PPPD and controls. We found worse performance in the virtual MWM spatial navigation test (12, 15–17) in patients presenting with PPPD than in both healthy subjects and patients suffering from other vestibular disorders but not presenting with PPPD.

## Materials and Methods

A cross-sectional study was conducted in which three groups of age-matched subjects were recruited: (i) patients suffering from PPPD, (ii) patients suffering from vestibular disorders other than PPPD, and (iii) healthy volunteers. Non-PPPD vestibular disorders included benign paroxysmal positional vertigo (BPPV), acute unilateral vestibulopathy, vestibular migraine and Ménière's disease. We chose to include these pathologies as representatives of the most common non-PPPD disorders in neuro-otology, producing different types of vestibular disfunctions. BPPV patients were analyzed before repositioning maneuvers. Ménière's and migraine patients were assessed during inter-ictal periods. Patients with acute vestibulopathy were assessed at least 3 months after onset, and only in patients who did no longer had spontaneous nystagmus, and that had not begun vestibular rehabilitation at the time of the procedures related to this research.

All subjects were assessed by means of a virtual version of the MWM task. Additionally, the Montreal Cognitive Assessment (MoCA) test was performed to exclude patients with cognitive impairment, which can affect the navigation assessment.

Patients being treated in an outpatient neurotology–otolaryngology unit at Clínica Alemana de Santiago medical center in Chile were invited to participate in the study. The study was performed in accordance with the Helsinki declaration. Written informed consent was obtained. This study is part of the “Characterization of Patients with Vestibular Disorders,” a larger research endeavor, which has been approved by Clínica Alemana de Santiago's Scientific Ethical Committee. We set the inclusion criteria to be participants aged between 18 and 65. This upper limit was particularly relevant to diminish the possibility of cognitive impairment affecting the navigation assessment. The exclusion criteria were patients with known psychiatric or neurological conditions in their medical records and patients with a MoCA score of <25 points.

Patients were assessed during the initial medical consultation for PPPD (1), as well as for other vestibular diseases, following the most recent Bárány Society diagnostic criteria for the definitive forms of the diseases (21–25). *Ad hoc* audiological and vestibular tests were administered, including pure tone audiometry, video-nystagmography, video head impulse, and vestibular evoked myogenic potential tests. During these tests, the virtual MWM test was conducted by an audiologist blinded to the current diagnosis of the patient, who also conducted MoCA.

**Figure 1:**
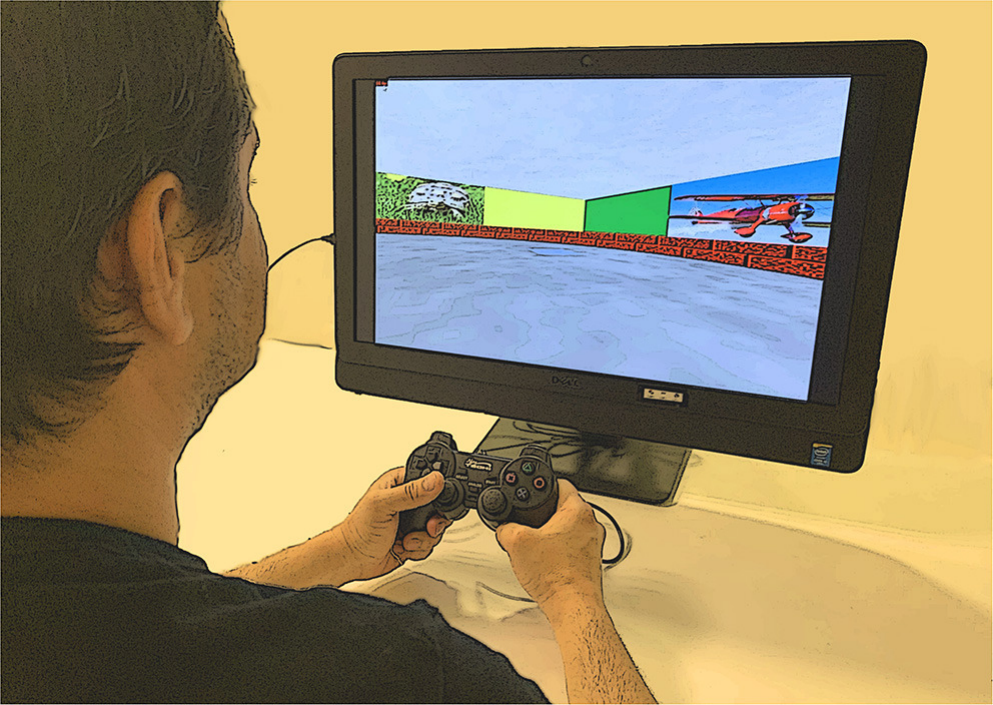
Experimental setting. All virtual Morris Water Maze tests were conducted in front of the same 24-inch desktop computer, and the subjects used a gaming joystick to navigate the virtual scenario.

**Figure 2:**
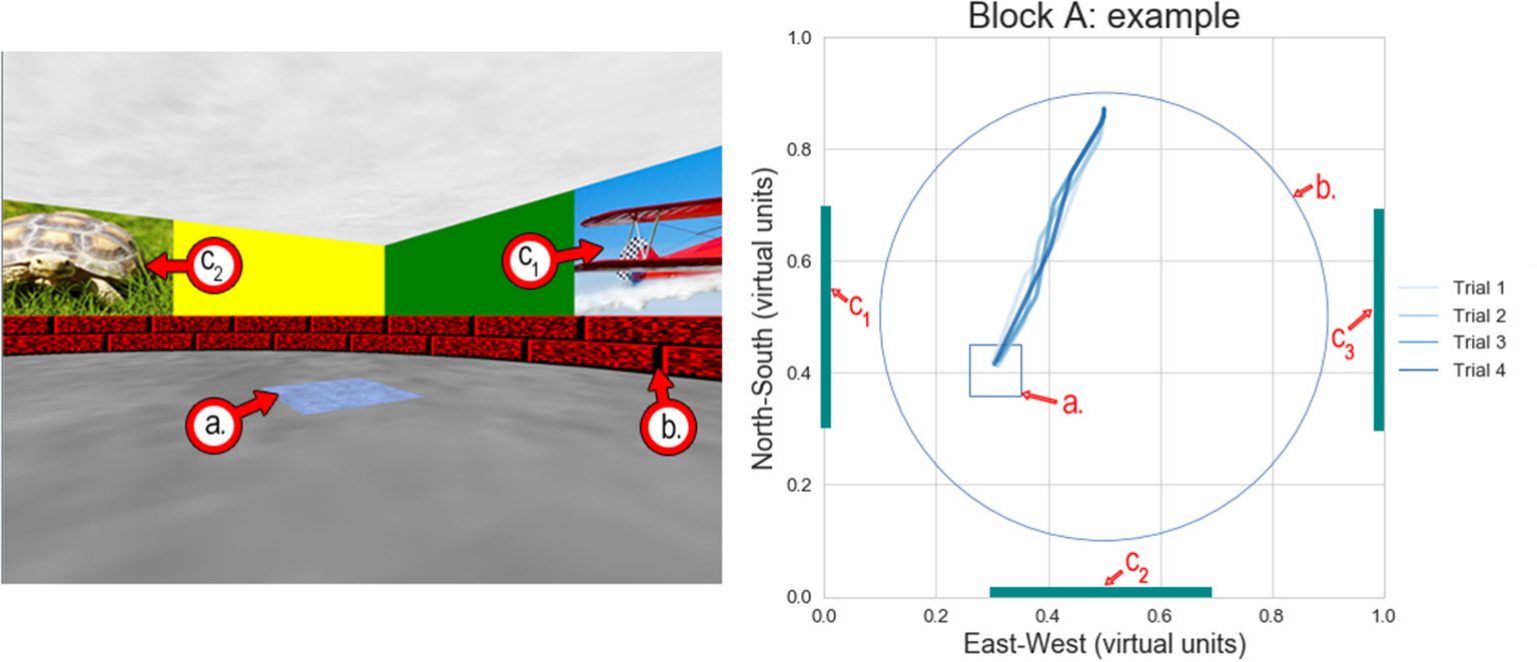
Example of the virtual Morris water maze test—Block A (fixed starting position/visible target). **(Left)** Screenshot of the virtual environment where the subjects were instructed to navigate toward the blue square on the ground. In the screenshot, the subject was in the northern half of the pool, looking southwards and slightly eastwards. **(Right)** Diagram showing the entire virtual environment from above. The four blue lines indicate the path the subject took from his starting point toward the target in each of the four replicate trials. The light blue line shows the first trial, and the dark blue line shows the last trial. In this case, the paths in every trial were almost identical, as the target was visible (a.) Square target—visible in this block. (b.) Boundary of the round pool in which the subjects were allowed to navigate. (c.) Visual cues on three of the four walls of the virtual environment. In all cases, C_1_ was an airplane, C_2_ was a turtle, and C_3_ was a flower.

All virtual MWM tests were conducted in front of the same 24-inch desktop computer, and the subjects used a gaming joystick to navigate the virtual scenario (Figure 1). This scenario was presented in a full screen by means of the software “Computer Generated Arena Software” (26) and consisted of a square room measuring 1 × 1 virtual units of distance in the “North–South” and “East–West” dimensions. Regarding the visual cues, in three of the four walls of the room, pictures that covered 0.4 virtual units of each wall were always centrally placed in the same relative order: the northern wall was empty, the eastern wall displayed a flower, the southern wall displayed a turtle and the western wall displayed an airplane. In the middle of the room, a round pool measuring 0.8 virtual units in diameter was placed (Figure 2).

The virtual MWM testing protocol consisted of 18 trials divided into three Blocks. Before each trial, patients could practice moving inside the round pool for as long as they needed in order to feel comfortable using the joystick to move in this virtual scenario; this practice session lasted between 2 and 5 min for all subjects.

In Block A (four trials), patients started at a fixed location, nearest to the northern end of the pool, facing southward. A fixed and visible blue square target area measuring 0.09 × 0.09 virtual units was placed on the ground in the southeast quadrant (Figure 2). Patients were instructed to navigate toward the blue target. When they arrived at the target, a rewarding sound was emitted, and the trial ended (each trial had a maximum duration of 1 min, even if the target was not reached). This first Block was designed to allow patients to become familiar with the virtual scenario and the testing protocol. Given the “training” nature of this Block, only four trials were conducted in this Block.

In Block B (seven trials), patients started at the same fixed location in the northern end of the pool. The square target was placed at a different fixed location (on the southwest quadrant) and was invisible. The target only turned visible when the patients stepped over it while navigating (and the rewarding sound was also emitted). Patients were instructed to navigate through the virtual pool to find the hidden target, and when they found the target, they had to remember its location to find it faster in subsequent trials. This process was repeated for seven trials.

In Block C (seven trials), patients started at a random location anywhere in the pool, facing toward a random direction. The square target was placed on a third different fixed location (in the east quadrant, slightly to the south) and was again invisible. The same instructions as those provided in Block B were given.

There are many ways to measure navigational performance in the MWM paradigm, including total path length traveled from the starting point to the target (measured in terms of “pool diameters,” which is equivalent to 0.8 virtual units in our study), latency or time spent navigating before reaching the target, and time spent in the quadrant where the target was located. Nevertheless, one of the most sensible metrics is Gallagher's proximity, or cumulative search error (CSE) (27–30). CSE is the sum of the average distance between the subject and the target at every second the subject is navigating during a single trial (28). Even when a subject is navigating in the vicinity of the target, if he or she does not find the target, he or she receives a lower CSE score than a subject who navigates far away from the target.

In addition, with the MWM paradigm, it is possible to assess not only the performance in a given trial but also spatial learning by analyzing performance across sequential trials. In the first trial (always depicted in the lightest blue color in the figures), the subjects navigated with the intent of locating the target. After the subjects found the target in one of the early trials of a given Block, they were expected to remember the target location and navigate more directly toward it in later trials, thus achieving better performance scores in later trials (darker blue routes on our diagrams).

Based on data in studies using a similar methodology (31, 32) and considering a minimal significant difference between groups of 4 pool diameters in terms of the CSE score, a statistical power of 80% and an alpha error of 5%, a sample size of 18 subjects per group was calculated. Given the small sample size, non-parametric tests were preferred in the comparative analysis between groups; the Kruskal–Wallis test was used to summarize the data within a distinct scenario, and the Friedman test was used when data of sequential trials in a given Block were assessed as repeated measurements. Receiver operating characteristic (ROC) curves were calculated with the clinical criteria for a PPPD diagnosis as the gold standard and CSE scores as discriminating values. Normalized kernel density estimation plots with standard distance deviations were calculated to reveal the areas of the pool most visited during navigation. All analyses and spatial drawings were conducted and constructed, respectively, with Python 3.6 statistical and graphical packages.

### Data Availability Statement

Data analyzed in this study is available at Dryad open repository.

## Results

Fifty-two patients were invited to participate in the study (2 declined, 50 accepted). A total of 19 patients fulfilling criteria for PPPD [as can be found on (1)] were recruited for the “PPPD” group, while 19 patients who did not meet the PPPD criteria but had other vestibular diagnoses were recruited for the “Vestibular” group (seven patients with PPPD, and five patients who could have joined the Vestibular group were excluded due to low MoCA results). BPPV, Ménières disease, vestibular migraine, and acute vestibulopathy were present in similar quantities in both (PPPD and non-PPPD) groups (Table 1). Eighteen healthy volunteers were included in the “Control” group. No significant differences were found in terms of age, sex, or MoCA results between groups. Table 1 summarizes the main characteristics of all subjects.

**Table 1:** Demographic summary of the PPPD, vestibular, and control groups.

		**Group**		
		**PPPD**	**Vestibular**	**Control**
Number of patients		19	19	18
Age	Median	47	46	43
	IQR^*^	32–58	28–53	34–56
	Range	22–65	23–62	29–60
Sex	Male/female	47.4/52.6%	42.1/57.9%	45/55%
MoCA	Median	26	27	28
	IQR	24–27	26–29	27–29
Diagnosis number [percentage of group]	BPPV^**^	3 [15.8%]	2 [10.5%]	–
	Vestibular migraine	6 [31.6%]	4 [21.1%]	–
	Menière's disease	5 [26.3%]	6 [31.6%]	–
	Acute unilateral vestibulopathy^***^	6 [31.6%]	8 [42.1%]	–

* *IQR, Interquartile range*.

** *BPPV, Benign paroxysmal positional vertigo*.

*** *Vestibular neuritis. More than 3 months after onset*.

*The sum of the diagnosis percentages is >100%, as some patients in both the PPPD and the vestibular groups presented more than one of the listed diagnoses. There were no differences in age, sex, or cognition between the three studied groups*.

**Figure 3:**
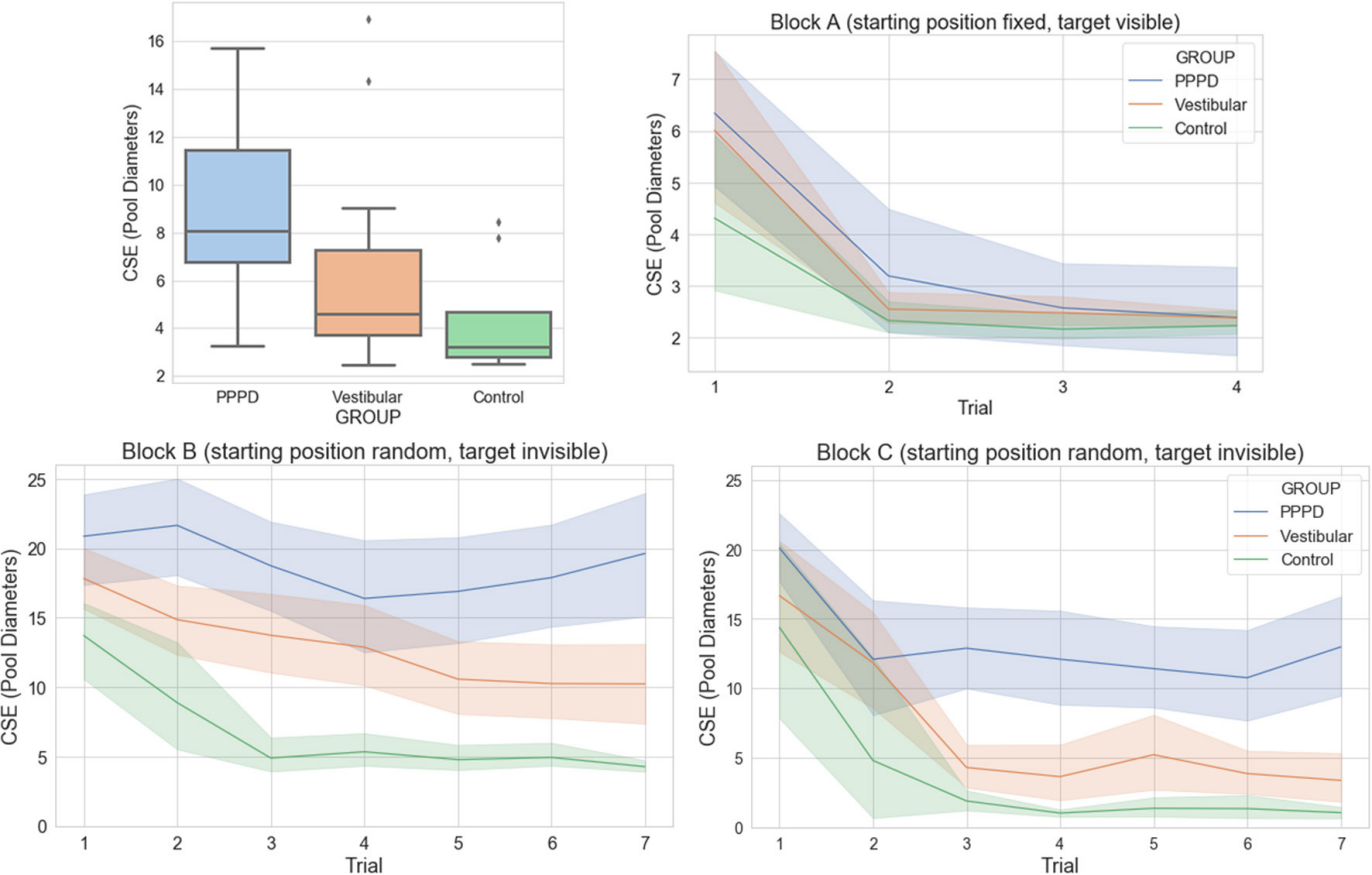
Cumulative search error (CSE) performance during MWM navigation in the PPPD, vestibular, and control groups. In all panels, a larger CSE reflects poorer performance in spatial navigation. **(Upper-left)** Boxplot of CSE performance for each group (PPPD group in blue, vestibular group in orange, controls in green), summarizing all 18 trials/three blocks. The difference between each group was significant (Kruskal–Wallis, *p* < 0.004; Dunn's *post-hoc* testing confirming the difference between every pair of groups). **(Upper-right)** Mean and standard error are shown for the CSE scores for each of the four trials in Block A (visible target) for each group. There were no significant differences between groups. **(Lower)** Mean and standard error are shown for the CSE scores for each of the seven trials in Block B **(lower-left)** and block C **(lower-right)** for each group. The Friedman test showed significant differences between each group across the seven trials in blocks B and C (*p* < 0.001).

In terms of path length, latency/time taken to reach the target, and CSE scores (considering all trials and blocks), the PPPD group performed worse than the patients in the vestibular and control groups did (*p* < 0.05, Kruskal–Wallis, Dunn's *post-hoc* test, see Table 2). We also found that the vestibular group performed worse than the healthy controls did in terms of the CSE scores. Figure 3 (upper left panel) shows the difference in the average CSE scores, including all trials between groups.

**Table 2:** Navigation performance after averaging all trials in the three blocks.

	**Group**				
	**PPPD**	**Vestibular**	**Control**	**Kruskal–Wallis *F*-value *p*-value**	***Post-hoc* Dunn's test (*p* < 0.05)**
Path length (pool diameters)	**2** Mean = 1.9 (SD = 1.4)	**1** Mean = 0.8 (SD = 1.4)	**1** Mean = 0.6 (SD = 0.9)	7.45 0.015	PPPD > Vestibular PPPD > Control
Time (seconds)	**20** (IQR = 16–35)	**14** (IQR = 12–21)	**10** (IQR = 9–17)	7.64 0.011	PPPD > Vestibular PPPD > Control
CSE (pool diameters)	**7** (IQR = 5–11)	**4** (IQR = 4–7)	**3** (IQR = 2–6)	9.21 0.004	PPPD > Vestibular PPPD > Control Vestibular > Control

*SD, standard deviation; IQR, interquartile range; CSE, cumulative search error*.

*Medians are shown in bold. In path length, means and SDs are added to better assess the differences among groups*.

When the CSE was analyzed within each block, all subjects, regardless of their group, performed equally well in Block A (fixed initial position and target visible). Figure 3 (upper right panel) shows this similarity between groups in each of the four trials in Block A (no significant difference was observed). In all cases, subjects navigated in a straight line from the starting point toward the target (as seen in Figure 2). Higher CSE scores were recorded only in trial 1 (the first time all subject attempted to complete the task of navigating toward the target).

On the other hand, an important difference was observed in Block B (starting position fixed, invisible target) and Block C (starting position random, invisible target). On both blocks, the PPPD group performed worse than the vestibular and control groups did, and the vestibular group performed worse than the control group did [Friedman test (*p* < 0.001), with Bonferroni *post-hoc* testing confirming that all groups were different from each other]. Moreover, in both navigationally challenging settings, the control, and vestibular groups showed successful spatial learning, as their CSE scores decreased across trials, while the PPPD group showed a lack of improvement in CSE scores in the later trials. Figure 3 (lower panels) presents these data graphically.

**Figure 4:**
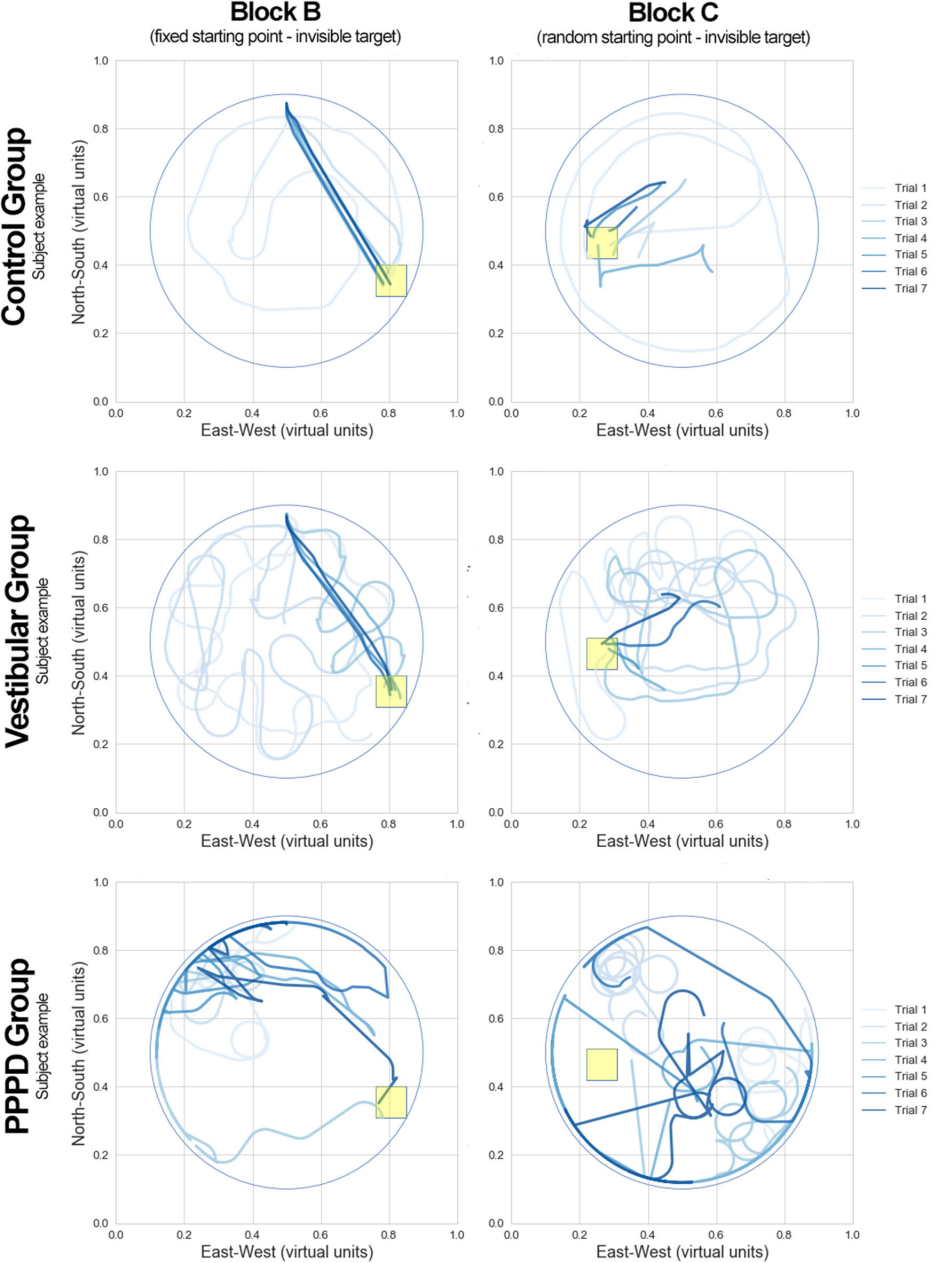
Examples of MWM navigation paths in the PPPD, vestibular, and control groups. Three individuals from each of the corresponding groups (control, vestibular, and PPPD) illustrating MWM performance in blocks B and C. All seven trials of a given block are drawn in the same diagram. Lines in a lighter blue color represent the early trials in each block, while the darker blue color represents the final trials. The yellow square box represents the target position in each block.

Figure 4 shows examples of the navigational performance during Blocks B and C of three individuals from the three different groups. In each diagram, all seven trials for each block are drawn. These plots illustrate how subjects from the vestibular and control groups learned these spatial tasks: once they found the hidden target, usually during the first three trials, they quickly learned its location and navigated in an almost straight line toward the target, even if the initial position was randomly set (Block C, diagrams to the right). The main difference between the control and vestibular groups is that the vestibular non-PPPD patients had slightly more difficulty in finding the target, as shown by the poorer CSE scores of this group depicted in Figure 3 (lower panels). On the other hand, subjects in the PPPD group behaved qualitatively differently. They explored the pool in a more disorganized fashion. While the PPPD group explored the pool by following many different routes, they failed to navigate the pool systematically; they neglected parts of the pool and left them unexplored. The PPPD group also tended to stay close to or continuously hit the wall of the pool and ventured less toward the center of the pool. Reaching the target in one of the trials did not improve the PPPD group's routes in subsequent attempts, showing that spatial learning is impaired in these individuals. Another repeatedly observed behavior was the tendency in many PPPD subjects to make circular movements, perhaps in an attempt to become familiar with the virtual scenario. These patterns were observed in at least half of the subjects in the PPPD group.

**Figure 5:**
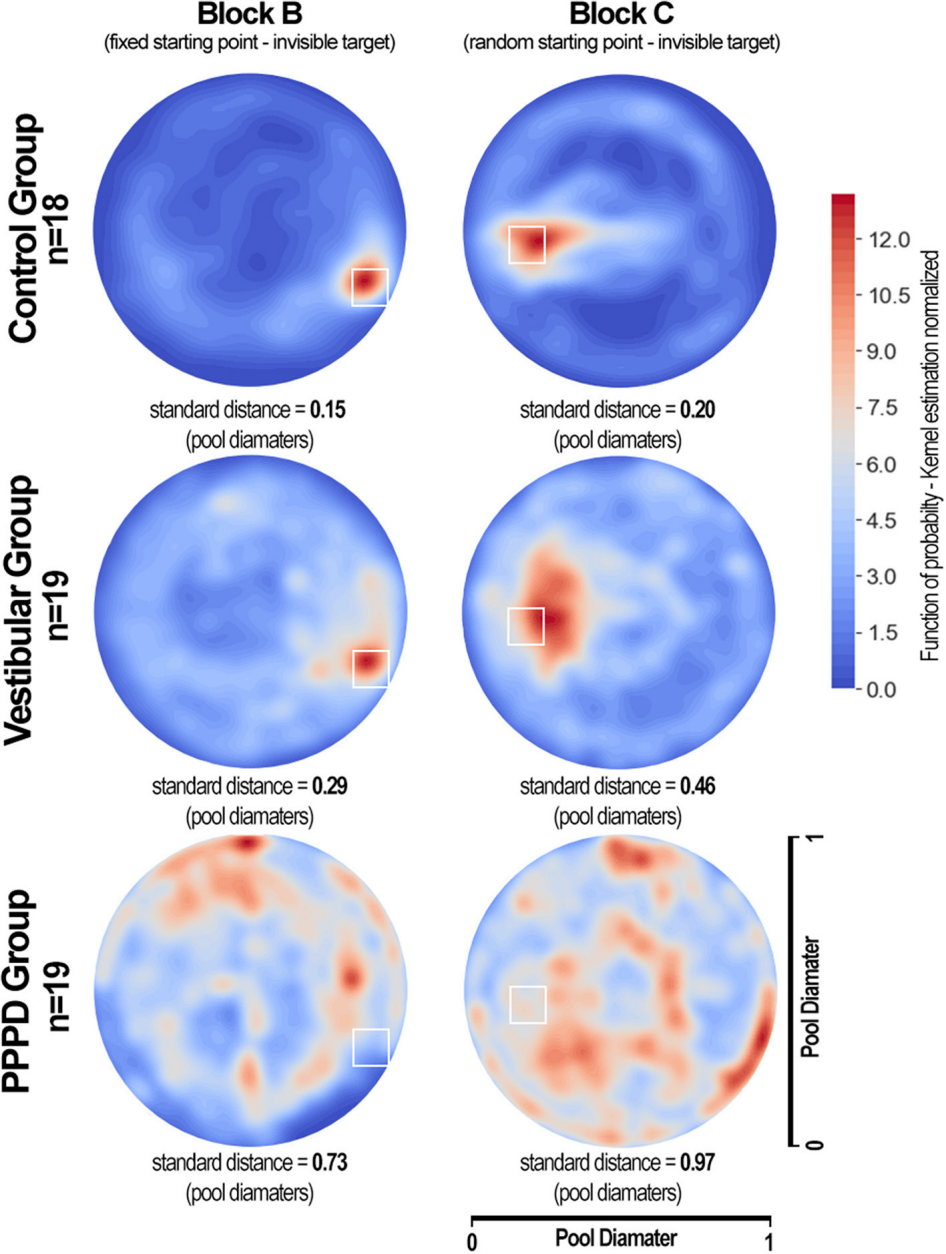
Density plots showing the areas that were most visited during MWM navigation in the PPPD, vestibular, and control groups. Each density plot presents data from all subjects in a given group in all seven trials of blocks B and C. The colored bar shows normalized values of density function (kernel estimation), with 50 levels of density colors (the sum of all values in the entire plot is equal to 1). The standard distance deviation for each group/block is shown. The areas in red represent the locations most visited during navigation, while the areas in blue represent the areas where subjects spent less time. The white squares represent the target in each block. Healthy volunteers in the control group navigated very close to the targets. Subjects in the vestibular group also concentrated their movements toward the targets, but with more dispersion. The PPPD group showed an inconsistent, more chaotically distributed density plot with a tendency to travel around the wall limits of the pool.

While the examples in Figure 4 are qualitatively striking, we sought to assess this difference in behavior at the group level (and not in individual cases). Therefore, to quantify the behavioral pathways in the MWM paradigm, we constructed density plots from kernel estimations (with normalization to 1 in each virtual pool and 50 distinct levels of probability), which described the position of the subjects from each group at every given moment in all trials in blocks A–C. Figure 5 shows these plots, where the red areas represent the portions of the pool with higher location probability (where the subjects spent more time), and the blue areas represent places where the subjects did not visit often or never visited. The white squares represent the target in each block. For each group/block pool, the standard distance deviation (the equivalent to standard deviation for two-dimensional spatial data) is shown as a numerical value of the amount of dispersion/concentration of the navigation patterns (higher values represent higher dispersion). Levene's test indicated the standard distance deviation to be significantly different between groups (W = 345.5; *p* < 0.001 for block B. W = 423.7; *p* < 0.001 for block C). Additionally, in the vestibular and PPPD patients, standard distance deviations were larger in block C than those in block B (W = 92.2, *p* = 0.21 for control; W = 198.2, *p* = 0.04 for vestibular; W = 339.2, *p* < 0.001 for PPPD).

**Figure 6:**
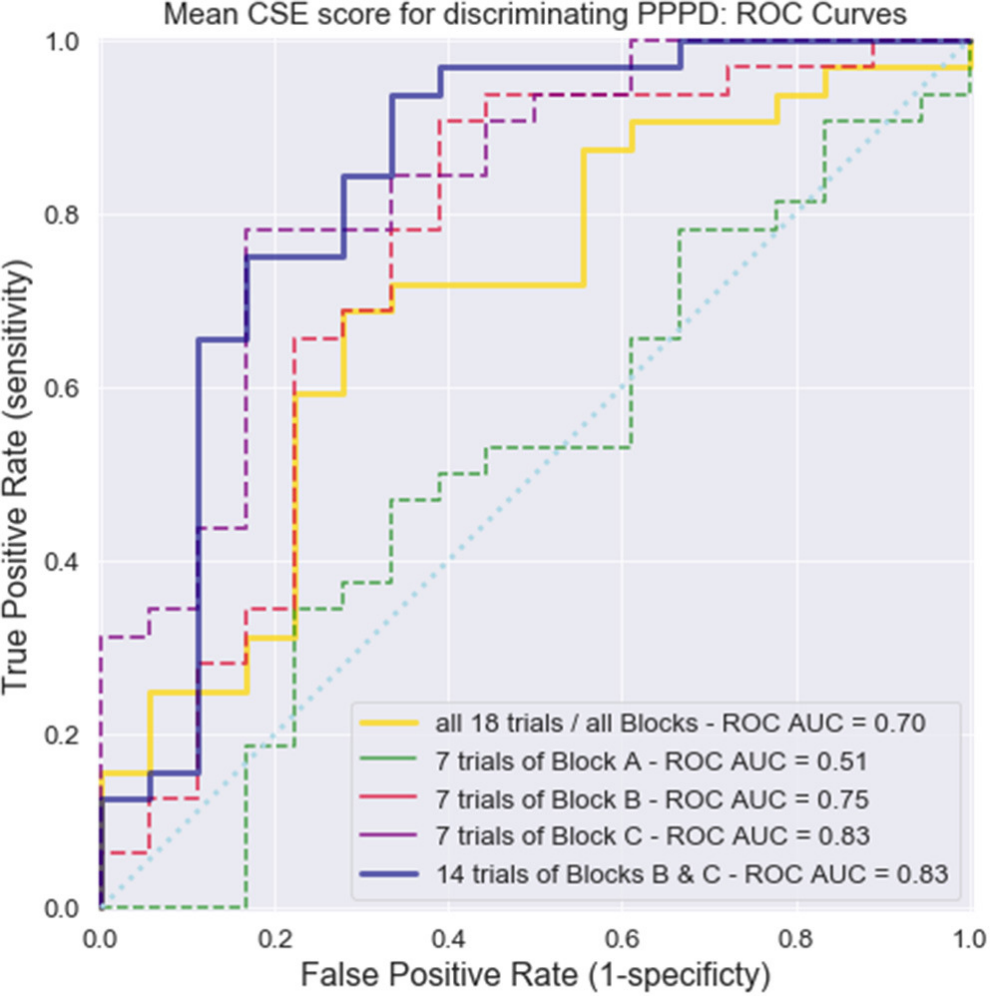
Receiver operating characteristics (ROC) curves for the diagnosis of PPPD using the MWM navigation task. Five ROC curves were calculated considering mean CSE scores as discriminating values and the presence of PPPD as the gold standard. Solid yellow line, all trials and all blocks together; dashed green line, Block A; dashed red line, Block B; dashed purple line, Block C; and solid blue line, Blocks B and C together (trials where the target was invisible, thus representing a navigational challenge). The area under the curve (AUC) values for each curve are shown.

Finally, Figure 6 shows the receiver operating characteristic (ROC) curves we plotted to assess whether the mean CSE scores could discriminate between PPPD and non-PPPD patients. Five distinct curves were calculated. The solid yellow line represents all 18 trials of the three blocks merged together, with an area-under-the curve (AUC) of 0.70. The dashed lines show curves for the mean CSE score of all four training trials with a visible target in Block A (in green, AUC = 0.51), all seven trials with invisible targets and fixed initial positions in Block B (in red, AUC = 0.75), and all seven trials with invisible targets and random initial positions in Block C (in purple, AUC = 0.83). The solid blue line represents the mean CSE performance of all trials where the target was invisible (Block B + C, non-training trials), with an AUC of 0.83.

In summary, we found that (i) in a non-navigationally challenging setting, i.e., that of Block A, all subjects performed equally well in terms of CSE (Figure 3—upper right panel); (ii) in navigationally challenging settings, vestibular patients performed worse than controls did, but PPPD patients performed even worse (Figure 3—upper left and lower panels); (iii) while the non-PPPD groups showed successful spatial learning, the PPPD patients showed a lack of change in the CSE scores across trials (Figure 3—lower panels); (iv) assessing all patients together—and not only individual examples—showed that the control and vestibular subjects focused their navigational movements near the target, while PPPD patients wandered all over the pool without a clear focus point (the amount of dispersion in this navigational pattern was larger in vestibular subjects than in controls, but it was even larger in PPPD patients); and (v) the CSE scores were able to distinguish PPPD patients from non-PPPD patients, particularly in the Block C setting, which yielded high AUCs in the ROC curves.

## Discussion

### Groups Did Not Differ in Performance When the Target Was Visible

Regardless of their group, all subjects performed equally well when asked to navigate toward a visible target (Figure 3—upper right panel). In MWM research, this kind of experimental setting that does not present a navigational challenge is intended to account for gross motor issues or problems with the handling of the computer elements of a virtual scenario task itself. Therefore, it is not surprising that CSE scores did not have sufficient power to discriminate between patients in block A and yielded an AUC of 0.51 (equal to chance), which is represented by the dashed green curve in Figure 6.

### Groups Performed Differently When Target Was Invisible. The PPPD Group Performed Worse Than the Non-PPPD Groups Did

The three groups in our study behaved differently in the navigationally challenging settings in Blocks B and C. In terms of total path length, latency (time), and particularly the CSE scores, PPPD patients showed a much worse performance in the MWM navigational task than did the age-matched vestibular patients and healthy controls. This finding can be clearly seen in Figure 3. Vestibular patients, particularly the PPPD patients, evidenced a significant impairment in spatial navigational abilities.

### The PPPD Group Showed Less Spatial Learning Than the Non-PPPD Groups Did

Spatial learning in an MWM setting includes the ability of subjects to identify a target in relation to environmental cues, retain this information in the form of a navigational map, and use this information to find more direct routes to the target in subsequent attempts. PPPD patients not only had lower CSE scores than the non-PPPD patients (control and vestibular groups) but also showed little spatial learning. As seen in Figure 3 (lower panels), the controls and vestibular subjects managed to improve their CSE scores in later trials, showing steep decreasing curves of the CSE scores. After the third or fourth trial, subjects showed a low “minimal” route, evidencing good spatial learning (particularly clear on Block C, presumably revealing cumulative learning of the whole experimental setting). The visual examples of individual performance shown in Figure 4 reinforce this idea. Routes in darker blue show how the selected control and vestibular subjects retained the location of the target and found a more direct path in subsequent trials. In contrast, PPPD patients showed less improvement or even no improvement in navigational performance across trials, as evidenced by the nearly flat curves in the lower panels of Figure 3. A selected case shown in Figure 4 helps us understand the behavior of PPPD subjects. Even after finding the target in an early trial, the subject did not navigate near that point and focused rather aimlessly in other parts of the pool, showing no evidence of remembering the location of the target. While Figure 4 presents a single case, the consistently high CSE scores across trials of the PPPD group reflects a lack of spatial learning in the group as a whole.

It remains to be seen in further studies if spatial learning remains impaired in PPPD in repetitions of such an experimental MWM testing over time (assessing how much navigation can be trained after repeating the task), or if this learning recovers after PPPD treatment.

### The PPPD Group Not Only Showed Worse Performance Than the Non-PPPD Groups Did but Also Showed Qualitatively Different Navigational Behavior

These results acquire much richer meaning after analyzing Figure 3 (individual examples) and Figure 4 (grand average) together. In contrast to healthy controls, vestibular patients not presenting PPPD had slightly more difficulties completing the task of navigating effectively to a hidden target. However, both healthy controls and non-PPPD vestibular patients explored the virtual pool with reasonable strategies, covering as much ground as possible, and when they find the target, they managed to locate it again in the later trials. This behavior is well-reflected by the concentration of subjects' movements near the target area in both non-PPPD groups, as shown in Figure 5.

PPPD patients not only wandered around far more than the non-PPPD subjects did while trying to find the target but also wandered in a very disorganized, non-strategic and disoriented fashion. This behavior can be observed in the selected PPPD examples in Figure 4, and it can be observed even more clearly in the widespread non-localized distribution of the grand average location probability of PPPD patients across the pool but not around the target, as shown in Figure 5, with significantly larger dispersion values.

### Spatial Navigation Impairment May Be a Key Feature of PPPD

Altogether, these findings suggest that navigation performance is impaired at some degree in all patients with vestibular impairment [which has been postulated in previous research (12, 13, 20)] and that this impairment is more severe in PPPD patients, to the point that it can be identified at the individual level and differentiate PPPD subjects from non-PPPD subjects (Figure 6). This ability of CSE scores to discriminate subjects was the highest in Block C (the most challenging navigational task in our experimental protocol, and thus the most sensitive task for identifying navigational impairment).

Again, this phenomenon appears to reflect not only the degree but also the quality of navigation in these patients. Figure 5 is particularly relevant for illustrating this point. While vestibular patients show significantly more disperse navigational patterns than controls do, both groups focus their navigational movements around the target. On the other hand, PPPD patients do not focus their movements around the target but rather move aimlessly around the pool, with much larger dispersion and more movements at the walls. This dispersion increases when the navigational setting is more demanding. This wandering without a clear effective navigational strategy or evidence of spatial learning can be further corroborated when reviewing individual cases such as those in Figure 4. Therefore, we believe the impairment or dysfunction of navigational abilities in PPPD patients to be a distinct one, different in degree and in qualitative features, in contrast to the lower-degree of impairment (and qualitatively normal navigational movements) presented in patients suffering from other vestibular disorders other than PPPD.

### Navigation Impairment Could Explain PPPD Symptomatology

We believe our finding raises the question of whether navigation-related functions in the brain represent a key feature in PPPD. We do not propose that PPPD patients have specifically and clinically relevant difficulties in navigating their everyday spaces and environments; instead, we propose that this disruption in the ability to construct, maintain and manage an internal map/model of the patients' spatial environment leads to an inappropriate and erroneous perception of the environment and therefore leads to the symptoms of PPPD. From this hypothesis, the latest definition of dizziness by the Bárány Society, which is “the sensation of disturbed or impaired spatial orientation without a false or distorted sense of motion,” gains new meaning (33).

While the relevance of vestibular input in the hippocampal navigational network has been studied, particularly its interaction with head direction cells (maintaining an internal “compass” of the direction relative to a determined “north” in the spatial environment), recent findings suggest that this computation is actually multimodal and fed strongly from extravestibular cues (visual, somatosensory, and efference copy computations) (34, 35).

From this perspective, it is easy to understand how a decrease in the quality of vestibular inputs (which can be supposed to have occurred in our vestibular group) can impair navigational performance. However, our findings of worse performance in the PPPD group can be interpreted as a disturbance not of the directly driven vestibular computations but rather of the extravestibular multimodal integrations, particularly given that head rotations are not used to aid navigation in our MWM setting. Future research should include comparisons of navigational performance in PPPD patients between a head-static setting (such as the one used in the present study) and an immersive virtual reality setting, where “real-life” head rotations contribute to orientation in the virtual space. We hypothesize that such a study would highlight PPPD disfunction as one of the computations of extravestibular cues.

### Brain Changes in PPPD: Is the Navigational Network Altered?

Following this line of thought, it would be very interesting to assess morphological changes in gray matter volume and connectivity—particularly in the hippocampus, entorhinal cortex, cerebellum and parietal regions—in these patients and the correlation between these changes and navigational performance. Recent imaging *has* found evidence of decreased volume and connectivity in many of these cerebral regions, as well as other regions, in PPPD patients (5, 36, 37). However, these changes have not been correlated with functional performance, such as navigation.

If our hypothesis that spatial navigation impairment is the core of PPPD is correct, one should expect to find worse spatial navigation performance in patients with less gray matter volume and connectivity in cortical areas specific to spatial navigation.

### A Diagnostic Tool?

From a more clinical and practical perspective, virtual MWM testing could eventually serve as a diagnostic tool, given that there are currently no biological markers for PPPD, and it remains an entity of pure clinical diagnosis. In our experimental setting, with a threshold of 8.16 pool diameters as the mean score of seven subsequent trials in the block C experimental setting, PPPD patients can be discriminated from non-PPPD patients with a sensitivity of 78.1% and a specificity of 83.3% (positive predictive value of 66%, negative predictive value of 88%). Such findings, particularly in patients with no other better explanation for a chronic vestibular syndrome (such as bilateral vestibulopathy, among others), could be strong objective indicators of PPPD. Nevertheless, this was not a study designed for testing a new diagnostic tool. Following diagnostic tests research (38), at the moment MWM could be considered as being in phase 1 (of 4) of becoming a reliable test.

### Limitations

Regarding the limitations of our study, we acknowledge that our findings must be confirmed in future research studies with larger sample sizes. Also, and given our present results, we believe necessary to compare PPPD navigational performance not with an “other vestibular disorders” group, but to every distinct neuro-otological disease by itself. If MWM could serve as diagnostic tool, it is very important to assess this test's behavior in patients presenting syndromes which can be easily confused in some cases with PPPD, such as Mal de Debarquement, visual induced dizziness, bilateral vestibulopathy, and vestibular migraine.

Follow-up or test-retest reliability would have also been of great assistance in determining the robustness of our findings. As has been commented further above, imaging techniques could have helped in understanding our findings more thoroughly, but also would have contribute to their validity by discarding any underlying neuroanatomical abnormalities.

Additionally, factors which could affect MWM performance could be even more rigorously controlled, including the use of more comprehensive neuro-psychological assessments for different degrees of cognitive impairment in addition to the MoCA, as tests assessing different psychological states, such as anxiety, which could be of influence at the time of testing. Given the high comorbidity rate of psychiatric and psychological disorders with PPPD, we acknowledge that this issue should be address in future research.

## Conclusions

Our findings suggest that while vestibular non-PPPD patients show poorer outcomes than healthy controls do in the navigational scores, PPPD patients perform significantly worse than both groups of individuals. PPPD navigational impairment is not only larger in magnitude when compared to that of non-PPPD subjects (vestibular and controls) but is apparently poorer in quality, showing disorganized and disoriented navigational patterns.

If confirmed in future studies, these findings highlight the relevance of a disturbance in navigation-related networks in the brain as a relevant feature in PPPD pathophysiology. Moreover, this distinctive spatial impairment in PPPD subjects might have a role as a biomarker for PPPD diagnosis.

## Data Availability Statement

The datasets generated for this study are available on request to the corresponding author.

## Ethics Statement

The studies involving human participants were reviewed and approved by Clínica Alemana de Santiago's Scientific Ethical Committee. The patients/participants provided their written informed consent to participate in this study.

## Author Contributions

HB designed and guided the study. HB, MC, JL, CG, LC, and KR contributed to patient's recruitment and assessment. HB and DM conducted statistical analysis. HB and PD wrote the manuscript.

### Conflict of Interest

The authors declare that the research was conducted in the absence of any commercial or financial relationships that could be construed as a potential conflict of interest.
